# *Anaplasma phagocytophilum* and *Babesia* Species of Sympatric Roe Deer (*Capreolus capreolus*), Fallow Deer (*Dama dama*), Sika Deer (*Cervus nippon*) and Red Deer (*Cervus elaphus*) in Germany

**DOI:** 10.3390/pathogens9110968

**Published:** 2020-11-20

**Authors:** Cornelia Silaghi, Julia Fröhlich, Hubert Reindl, Dietmar Hamel, Steffen Rehbein

**Affiliations:** 1 Institute of Comparative Tropical Medicine and Parasitology, Ludwig-Maximilians-Universität München, Leopoldstr. 5, 80802 Munich, Germany; froehlichejulia@yahoo.de; 2 Institute of Infectology, Friedrich-Loeffler-Institut, Südufer 10, 17493 Greifswald Insel Riems, Germany; 3 Tierärztliche Fachpraxis für Kleintiere, Schießtrath 12, 92709 Moosbach, Germany; praxis@dr-reindl.de; 4 Boehringer Ingelheim Vetmedica GmbH, Kathrinenhof Research Center, Walchenseestr. 8-12, 83101 Rohrdorf, Germany; dietmar.hamel@boehringer-ingelheim.com (D.H.); steffen.rehbein@boehringer-ingelheim.com (S.R.)

**Keywords:** tick-borne pathogens, cervids, *Capreolus capreolus*, *Dama dama*, *Cervus nippon*, *Cervus elaphus*

## Abstract

(1) Background: Wild cervids play an important role in transmission cycles of tick-borne pathogens; however, investigations of tick-borne pathogens in sika deer in Germany are lacking. (2) Methods: Spleen tissue of 74 sympatric wild cervids (30 roe deer, 7 fallow deer, 22 sika deer, 15 red deer) and of 27 red deer from a farm from southeastern Germany were analyzed by molecular methods for the presence of *Anaplasma phagocytophilum* and *Babesia* species. (3) Results: *Anaplasma phagocytophilum* and *Babesia* DNA was demonstrated in 90.5% and 47.3% of the 74 combined wild cervids and 14.8% and 18.5% of the farmed deer, respectively. Twelve *16S rRNA* variants of *A. phagocytophilum* were delineated. While the infection rate for *A. phagocytophilum* among the four cervid species was similar (71.4% to 100%), it varied significantly for *Babesia* between roe deer (73.3%), fallow deer (14.3%), sika deer (27.3%) and red deer (40.0%). Deer ≤2 years of age tested significantly more often positive than the older deer for both *A. phagocytophilum* and *Babesia* species. (4) Conclusions: This study confirms the widespread occurrence of *A. phagocytophilum* and *Babesia* species in wild cervids and farmed red deer in Germany and documents the co-occurrence of the two tick-borne pathogens in free-ranging sika deer.

## 1. Introduction

Because of their zoonotic potential, tick-transmitted pathogens such as the intracellular bacterium *Anaplasma phagocytophilum* and piroplasms of the genus *Babesia* have received substantial interest in both human and veterinary medicine in the past decades. The main vector of these pathogens in Europe is *Ixodes ricinus*, the most common and widely distributed hard tick [1]. *Anaplasma phagocytophilum* is one of the most frequently diagnosed tick-borne pathogens in humans in the USA but has rarely been found in humans in Europe, even though prevalence rates in ticks can be high [2]. Several cases of human babesiosis caused by *Babesia divergens* have been diagnosed in Europe [3], and infections caused by *Babesia microti* and *Babesia* sp. EU1 (referred to also as *Babesia venatorum*) have been reported [4,5]. 

Wild cervids play an important role in the transmission cycles of these tick-borne pathogens, as they are important hosts for feeding the vector ticks and are considered as a potential reservoir for the pathogens [6]. For these reasons, there is continuing interest in studying the prevalence of these tick-borne pathogens in wild cervids. In Europe, *A. phagocytophilum* DNA was found in many wild ungulate species [2]. In Germany, several variants of *A. phagocytophilum* have been identified in roe deer (*Capreolus capreolus*), fallow deer (*Dama dama*) and mouflon (*Ovis musimon*), a wild bovid ungulate [7,8]. Similarly, several species or genetic variants of *Babesia* have been detected in wild ungulates in Europe, including *Babesia capreoli, B. divergens, Babesia bigemina, Babesia motasi, Babesia ovis, Babesia pecorum*, *Babesia* sp. EU1, *Babesia odocoilei*-like, *Babesia* sp. MO1 and *Babesia* sp. CH1 [7,8,9,10,11,12,13,14,15,16,17]. Regarding the babesias of wild ungulate hosts in Germany, *B. capreoli*, *Babesia* sp. EU1 and *B. odocoilei*-like have been identified in roe deer, fallow deer and mouflon [7,8,9]. Knowledge of the clinical relevance of infection with *A. phagocytophilum* and/or babesias in wild ungulates remains incomplete; however, clinical signs or even death attributable to infection with these pathogens have been rarely reported [18,19,20].

As there are just a few published studies investigating the two pathogens concurrently in wild deer in general, especially in sika deer (*Cervus nippon*), and comparative studies in multi-species deer communities are scarce, the aim of the present work was to describe the occurrence of *A. phagocytophilum* and *Babesia* species in four species of cervids inhabiting the same area. In addition, red deer from a deer farm located in the area were examined. While roe deer and red deer (*Cervus elaphus*) are native to Germany, fallow deer have been naturalized several centuries ago, and sika deer were allowed to establish free-ranging populations in Germany only approximately 70 years ago [21]. However, in the area of investigation, the occurrence of both fallow deer and sika deer is related to the migration of these cervids from Bohemia, Czech Republic, into Bavaria, Germany, after the fall of the Iron Curtain in the 1990s [22,23,24,25,26].

Therefore, for the first time, we studied the presence of *A. phagocytophilum* and babesias in deer of four species (roe deer, fallow deer, sika deer and red deer) sharing one habitat in southeastern Germany and in red deer from a deer farm located within the same area. In addition, *A. phagocytophilum* variants based on the variation in the partial *16S rRNA*-gene and the diversity of *Babesia* species were studied.

## 2. Results

Out of the 101 deer examined, 77 (76.2%) yielded evidence of infection with *A. phagocytophilum* and/or *Babesia* species: 68 (91.9%) of the combined 74 wild deer (29/30 roe deer, 96.7%; 5/7 fallow deer, 71.4%; 19/22 sika deer, 86.4%; 15/15 red deer, 100%) and nine of the 27 farmed red deer (33.3%) tested positive. 

### 2.1. Anaplasma phagocytophilum

*Anaplasma phagocytophilum* DNA was found in the spleen tissue of 67 out of the 74 combined wild cervids (90.5%) and four of the 27 (14.8%) farmed red deer (*p* < 0.0001). There was no significant difference in the infection rate between wild roe deer (28/30, 93.3%), fallow deer (5/7, 71.4%), sika deer (19/22, 86.4%) and red deer (15/15, 100%) (*p* = 0.1416). However, *A. phagocytophilum* DNA was detected significantly more often in wild red deer than in farmed red deer (15/15 vs. 4/27; *p* < 0.0001). The overall rate of *A. phagocytophilum* infection did not differ between male and female combined wild cervids (39/43, 90.7% vs. 28/31, 90.3%; *p* = 1.0). There was a significant association of the age of the combined wild cervids and the rate of *A. phagocytophilum* infection, with 100% (8/8), 97.5% (39/40) and 61.5% (16/26) of the animals <1 year, 1 to 2 years or > 2 years of age, respectively, testing positive (*p* = 0.0002). Overall, DNA of *A. phagocytophilum* was more frequently detected in young deer of up to 2 years of age than in the older animals (47/48 vs. 16/26; *p* < 0.0001). The analysis of the *A. phagocytophilum*-positive samples for variation in the partial *16S rRNA* gene resulted in the delineation of 12 variants (Table 1).

### 2.2. Babesia Species

DNA of *Babesia* species in spleen tissue was found in 35 out of 74 combined wild cervids (47.3%) and five of the 27 (14.8%) farmed red deer (*p* = 0.0111). Infection rate varied significantly (*p* = 0.0018) among the wild cervids (roe deer: 22/30, 73.3%; fallow deer: 1/7, 14.3%; sika deer: 6/22, 27.3%; and red deer: 6/15, 40%), with roe deer found to be more frequently infected with babesias than fallow deer, sika deer and red deer (*p* < 0.05). There was no significant difference in the *Babesia* species rate of infection between wild and farmed red deer (6/15 vs. 5/27; *p* = 0.1582). The overall rate of *Babesia* species infection did not differ between male and female combined wild cervids (19/43, 44.2% vs. 17/31, 54.8%; *p* = 0.4801). There was a significant association of the age of the combined wild cervids and the rate of *Babesia* species infection, with 62.5% (5/8), 57.5% (23/40) and 26.9% (7/26) of the spleen samples of animals <1 year, 1 to 2 years or >2 years of age, respectively, testing positive (*p* = 0.0306). Overall, DNA of *Babesia* species was significantly more frequently detected in wild young deer of up to 2 years of age than in the older animals (31/48 vs. 7/26; *p* = 0.0144).

Sequencing of the 40 *Babesia*-positive samples revealed:

- 18 sequences of *B. capreoli* from roe deer, with 99%–100% similarity to other GenBank^®^ entries for *B. capreoli* from roe deer from Germany (e.g., accession number KU351826; [8].
- Three sequences of *Babesia* sp. EU1 from roe deer, with 100% identity to GenBank^®^ entries for *Babesia* sp. EU1 from *I. ricinus* from Spain (KM289158), *Ixodes persulcatus* from Mongolia (LC005775) and reindeer (*Rangifer tarandus*) from Germany (KM657247).
- Eight sequences of *B. odocoilei*-like from three sika deer and five red deer (four wild deer, one farmed deer) showing 99–100% similarity to sequences found in fallow deer in Germany (KU351828) and in red deer in Austria (JN543180).
- Eight sequences from two sika deer and six red deer (two wild deer, four farmed deer) with 100% identity to *B. divergens* from *I. ricinus* in Poland (KU748896) and red deer in Austria (KX018019). In addition, sequences obtained from one fallow deer and one sika deer were closely related to both *B. capreoli* and *B. divergens* but did not allow for discrimination of the two species, and the sequence from a roe deer was short and could only be identified to *Babesia* genus level.

No case of mixed *Babesia* species infection was detected.

Exemplary sequences of this study (representing all *Babesia* species in the different animal species) were deposited in GenBank^®^ database under accessions numbers MT151374-MT151379. 

### 2.3. Anaplasma phagocytophilum/Babesia Species Co-Infections

DNA of both *A. phagocytophilum* and *Babesia* species was detected in 34 of the 74 (40.5%) combined wild cervids but in none of the 27 farmed red deer (*p* < 0.0001). *Anaplasma phagocytophilum*/*Babesia* species co-infection rate varied significantly (*p* = 0.0039) between wild roe deer (21/30, 70.0%), fallow deer (1/7, 14.3%), sika deer (6/22, 27.3%) and red deer (6/15, 40%). Roe deer tested positive more frequently for co-infection than fallow deer (*p* = 0.0054) and sika deer (*p* = 0.0045), and there was no significant difference in the rate of co-infection between roe deer and red deer (*p* = 0.1049). Among the combined wild cervids, overall *Babesia* species infection was associated with the *A. phagocytophilum* infection (*Babesia*-positive/*A*. *phagocytophilum*-positive: 34/67, 50.7% vs. *Babesia*-positive/A. *phagocytophilum*-negative: 1/7, 14.3%; *p* < 0.0001).

## 3. Discussion

Despite the growing knowledge on the role of wild (free-ranging) ungulate animals in the epidemiology of (potential) zoonotic tick-transmitted infections, there are few comparative studies on different species of deer in natural multi-species deer communities. Results of the present study demonstrate that infections with the two *I. ricinus*-transmitted pathogens, *A. phagocytophilum* and *Babesia* species, are common in the sympatric roe deer, fallow deer, sika deer and red deer in the Upper Palatinate Forest. 

### 3.1. Anaplasma phagocytophilum 

The infection rate in roe deer obtained in this study (93.3%) was similar to infection rates previously reported in roe deer from Germany (consistently >90%) but was higher than rates reported in studies from other countries in Europe, i.e., Hungary, UK, Austria, Italy and Spain [2,7,8,27,28,29,30]. The *A. phagocytophilum* infection rate found in fallow deer was similar to that reported in previous studies from Italy, Germany and Hungary, which were around 70% [8,29,31]; however, no *A. phagocytophilum* DNA was isolated from spleen tissue of six adult fallow deer bucks and one male fawn from one location in Austria [32]. The infection rate in the wild red deer in this study (100%) was higher than that found in most previously reported studies in wild red deer [2] but similar to recently reported work from Hungary [29]. Similar to sika deer from Japan [33], *A. phagocytophilum* DNA has been detected at a high rate for the first time in wild sika deer in the Upper Palatinate Forest in Germany (96.3% and 86.4%, respectively). Phylogenetic studies on isolates of sika deer from Japan, which were earlier identified as *A. phagocytophilum*, have later demonstrated them to be distinct from *A. phagocytophilum* [34]. The respective *Anaplasma* was named *AP*–sd and was found with prevalences up to 51% in sika deer in Japan [35]. In Europe, *A. phagocytophilum* was previously detected in the blood of 11 of 32 farmed sika deer in Poland [36] and 6 of 12 sika deer from the New Forest, UK [30]. However, another study in farmed sika deer in China could not detect DNA of *A. phagocytophilum* in the 68 investigated animals [37]. There were no significant differences in prevalence rates between the sympatric species of wild cervids in this study. A study in Hungary found red deer infected significantly more often than fallow deer and roe deer [29]. However, in the present study, all cervid species tested significantly more often positive for *A. phagocytophilum* than the red deer from the deer farm, which is in line with the findings of a study conducted in Poland comparing infection rates determined in free-living and farmed red deer [36]. There were no differences in the infection rates with *A. phagocytophilum* of male and female deer which is consistent with previous studies [7,8,27]. In agreement with previously reported studies [8,27], the present study showed that animals older than 2 years tested positive less frequently than the younger animals. However, no infection-rate-to-age relationship was found in another study conducted in southern Germany [7]. Overall, the results of the present study support the hypothesis that roe deer and other cervid species represent the main reservoirs for this tick-borne pathogen in Central Europe and emphasize the role of cervids in hosting ticks.

### 3.2. Anaplasma phagocytophilum Variants

Twelve variants of the partial *16S rRNA* gene of *A. phagocytophilum* were delineated in this study. Variant B has been previously described as the prototype variant of the Human Granulocytic Anaplasmosis agent (GenBank^®^ accession numbers: U02521). It has also been found in dogs and horses with granulocytic anaplasmosis [38,39] and was detected in several species of wild ungulates [8,27]. In the present study, this variant was detected in sika deer, which provides further evidence that variant B is infectious to a broad range of cervid and bovid hosts. Variant W, the agent of tick-borne fever in sheep and cattle in Europe [40,41], was found in all species of cervids in this study. Variant S was previously found in several wild ruminants and in dogs (FJ829790) and horses (JF893934) with granulocytic anaplasmosis [8,27,38,39]. The potentially apathogenic variants X and Y. as well as the potentially wild ruminant specific variants I and V, were previously detected in roe deer and mouflon [7,8,27] and were demonstrated in sika deer and fallow deer in this study. Five further variants were found in single cases only. Separation of *A. phagocytophilum* into genetic lineages associated with roe deer and/or other wild ruminants, domesticated animals or generalist variants have been discussed previously [28,39,42,43,44].

### 3.3. Babesia Species 

This study shows that in one geographic location, all together at least four babesias are circulating among the wild cervid species. The overall prevalence of *Babesia* spp. in wild cervids of 47.3% is comparable to the results of other studies in wild ungulates in Europe [8,10,11,15,45]. The *Babesia* infection rate in roe deer in the present study was within the range previously reported from roe deer in Germany [7,8,12]. It was significantly higher than the *Babesia*-positivity found in the sympatric fallow deer, sika deer and red deer and hence in line with the findings of a previous study from Germany that found roe deer. Overall, these findings support the hypothesis that roe deer may be a natural reservoir for several *Babesia* species, especially for *B. capreoli,* which represents the most common *Babesia* species identified in roe deer in this study and in previous studies from Germany [7,8]. For the first time, we report the detection of babesias in sika deer in Germany. Sika deer in Japan showed a very high *Babesia* prevalence in a PCR-Reverse Line Blot approach (94.4%, with a catch-all probe; [46]), while the infection rate in farmed sika deer found in a study in China (35.3%; [47]) was comparable to the results of the analysis of sika deer samples in the present study. However, the babesias described from farmed sika deer in China [47,48,49]) and from wild sika deer in Japan [50] were distinct from those detected in sika deer in the present study. In Europe, *B. capreoli* was previously described to infect sika deer/red deer hybrids in Ireland [51]. The identification of *Babesia* sp. EU1 in roe deer in the Upper Palatinate Forest confirmed the results of previous studies in Germany [7,8]. *Babesia odocolei*-like babesias, which were previously isolated from fallow deer in Germany [8], were reported to infect sika deer and red deer in Germany for the first time. In addition, for the first time in Germany, the present study documented the occurrence in sika deer and red deer of *B. divergens*. Consistent with previous studies [7,8,12], there was no association between *Babesia* infections and the gender of the animals. Similar to the *A. phagocytophilum* infection, younger animals tested positive significantly more often for *Babesia* than the older animals in the present study. This inverse relationship has been reported previously [12]; however, other studies did not find a correlation between the rate of *Babesia* infection and the age of the animals [7,8]. As type of sampling, sample size and composition of the sample set per cervid species investigated in this study reveal considerable variability, larger sample sizes or meta-analysis of data from various studies should be considered to allow for more conclusions as to the potential association of the prevalence of infections and the gender and age of the wild ruminants.

### 3.4. Co-Infections

In the present study, *A. phagocytophilum* and *Babesia* spp. co-infections were recorded in 40.5% of the combined wild cervids, including all four species of cervids. Examining roe deer and fallow deer from several locations in Germany, a co-infection rate of 50% was found in the combined cervids, with roe deer outnumbering fallow deer (60.8% and 11.6%, respectively; [8]). In line with the findings in the present study (70%), roe deer consistently demonstrated high rates of *A. phagocytophilum* and *Babesia* species co-infections (88.4% [7]; 60.8% [8]). In agreement with previous studies in wild ruminants in Germany and in Austria [7,8,12], results of this study strongly support the hypothesis of the crucial role of roe deer as a major host for the two tick-borne pathogens. The statistically significant association of *A. phagocytophilum* and *Babesia* species documented in this study confirms the findings of earlier studies [8,12]. 

## 4. Materials and Methods 

**Figure 1:**
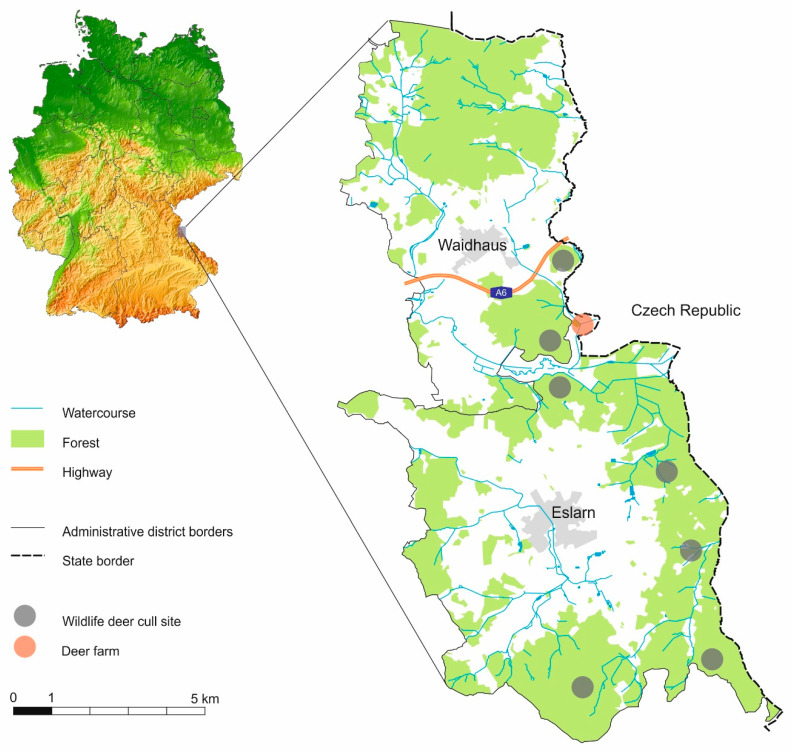
Sampling location in the Upper Palatinate Forest, Bavaria, Germany.

Spleen samples of 74 wild (free-ranging) cervids comprising 30 roe deer, 7 fallow deer, 22 sika deer, 15 red deer) from one geographical area at a total of 7 sites in the Upper Palatinate Forest, Bavaria, close to the border to the Czech Republic, and 27 red deer from a deer farm located within that area [52,53] were collected. The spleen was separated from the gastrointestinal tract of the culled animals, and one approximately 1 cm wide and 5 cm long strip of tissue from the margin of each spleen was collected using disposable scalpels. The spleen tissue was preserved in 15 mL Falcon tubes filled with 70% ethanol. The wild deer were harvested within a radius of less than 10 km of the deer farm (Figure 1) during the hunting season spanning the months of May to February in the years of 2011 to 2014; the farmed red deer were culled during the months of November and December 2012 and January 2013. All samples were collected from animals harvested by regular hunting (hunting during the regular hunting season) and were considered clinically unsuspicious based on observations of the live animals before culling and gross inspection by the hunters. The age of the animals was estimated by the hunters based on gross morphological characters.

Information on the sex and age (stratified into three categories) structures of the deer are summarized in Table 2. The spleen tissue (approximately one cm^3^ per animal) was stored in 15 mL Falcon tubes filled with 70% ethanol until analysis. DNA extraction from the spleen tissue was carried out with the High Pure PCR Template Preparation Kit (Roche, Mannheim, Germany) according to the manufacturer’s instruction for animal tissue as previously described [27]. Quantity and quality of extracted DNA were tested with a spectrophotometer (NANODROP^®^ ND-1000, Peqlab. Erlangen, Germany). All samples were screened for the presence of DNA of *A. phagocytophilum* with a real-time PCR targeting a 77 base pairs (bp) part of the *msp2* gene, and for *A. phagocytophilum*-positive samples, a PCR targeting of a 497 bp part of the *16 S rRNA* gene was performed [8,54,55].

*Babesia* DNA was detected with PCR targeting a part of the *18S rRNA* gene [8,56]. The amplified part of the gene includes two out of three base positions (at positions 631 and 663 of the full gene), which were described previously as discriminating between *B. capreoli* and *B. divergens* [19]). Amplicons of the conventional PCRs were visualized by gel electrophoresis [8]. As positive controls, genomic DNA extracted from *A. phagocytophilum* cell culture and from *B. microti* from a continuous culture in BALB/c mice (courtesy of. E. Siński) were used in each PCR run. Molecular grade water was used as negative controls. PCR products were purified with QIAquick PCR Purification Kit (Qiagen, Hilden, Germany) and sequenced externally (Eurofins MWG Operon, Ebersberg, Germany). Sequences were analyzed and compared with sequences available from GenBank^®^ with the BLASTn tool of the National Center for Biotechnology (http://blast.ncbi.nlm.nih.gov/Blast.cgi) as previously described [8]. 

Statistical analysis was performed using VassarStats^®^ (http://vassarstats.net/). Associations between positivity for infection and variables representing host demographic factors (age group, sex) and origin (subsets (combined) “wild” or “farmed”) were assessed using contingency tables and Fisher’s exact test. Age and sex were considered for combined wild cervids only because the number of animals per species and composition varied for the four cervids (Table 1). The association between the presence of *Babesia* species and *A. phagocytophilum* was analyzed using McNemar’s test for matched-pair samples. All testing was two-tailed, and level of significance for all analyses was set at *p* < 0.05. 

## 5. Conclusions

This study confirms the widespread occurrence and diverse nature of tick-transmitted *A. phagocytophilum* and *Babesia* species infecting sympatric cervids in Germany and provides data on both *A. phagocytophilum* and *Babesia* species in free-ranging sika deer in Europe. It documents the co-occurrence of both pathogens in sika deer. Additionally, this study provides further evidence for the occurrence of *B. odocoilei*-like babesias in cervids in Europe.

## Figures and Tables

**Table 1:** Variants of the partial *16S rRNA* gene of *Anaplasma phagocytophilum* isolated from sympatric cervids from one location in the Upper Palatinate Forest, Germany.

Variant	Number of Variants					GenBank^®^ Accession Numbers
	Total	Roe Deer	Fallow Deer	Sika Deer	Red Deer	
B	5	0	0	3	2	KU705180, KU705181, KU705133, KU705136
I	7	6	0	1	0	KU705140, KU705141, KU705184, KU705152, KU705154, KU705158, KU705160
J	1	0	0	1	0	KU705187
S	9	0	0	4	5	KU705178, KU705182, KU705124, KU705128, KU705186, KU705189, KU705131, KU705134, KU705138
V	2	1	1	0	0	KU705198, KU705153
W	13	2	1	4	6	KU705176, KU705177, KU705179, KU705125, KU705127, KU705129, KU705145, KU705197, KU705185, KU705132, KU705135, KU705137, KU705156
X	8	6	1	1	0	KU705142, KU705146, KU705188, KU705149, KU705150, KU705155, KU705159
Y	5	5	0	0	0	KU705143, KU705144, KU705148, KU705151, KU705157
16S-24	1	1	0	0	0	KU705147
16S-25	1	0	0	0	1	KU705130
16S-26	1	0	0	0	1	KU705139
16S-30	1	0	0	0	1	KU705126
**Total**	**54**	**21**	**3**	**14**	**16**	---

**Table 2:** Cervids sampled in one geographic location in the Upper Palatinate Forest, Germany for analysis of infection with *Anaplasma phagocytophilum* and *Babesia* species.

Cervid Species and Origin	Number of Deer Sampled, Total (Male, Female)	Age Group, Total (Male, Female)		
		<1 Year	1–2 Years	>2 Years
Roe deer, wild	30 (9, 21)	2 (1, 1)	22 (6, 16)	6 (2, 4)
Fallow deer, wild	7 (7, 0)	0	3 (3, 0)	4 (4, 0)
Sika deer, wild	22 (17, 5)	0	13 (11, 2)	9 (6, 3)
Red deer, wild	15 (10, 5)	6 (5, 1)	2 (1, 1)	7 (4, 3)
Combined wild deer	74 (43, 31)	8 (6, 2)	40 (21, 19)	26 (16, 10)
Red deer, farmed	27 (17, 10)	25 (17, 8)	0	2 (0, 2)
Total deer	101 (60, 41)	33 (23, 10)	40 (21, 19)	28 (16, 12)

## References

[1] Rizzoli A., Silaghi C., Obiegala A., Rudolf I., Hubálek Z., Földvári G., Plantard O., Vayssier-Taussat M., Bonnet S., Spitalská E. (2014). *Ixodes ricinus* and its transmitted pathogens in urban and peri-urban areas in Europe: New hazards and relevance for public health. Front. Public Health.

[2] Stuen S., Granquist E.G., Silaghi C. (2013). *Anaplasma phagocytophilum*—A widespread multi-host pathogen with highly adaptive strategies. Front. Cell Infect. Microbiol..

[3] Hildebrandt A., Gray J.S., Hunfeld K.P. (2013). Human babesiosis in Europe: What clinicians need to know. Infection.

[4] Häselbarth K., Tenter A.M., Brade V., Krieger G., Hunfeld K.P. (2007). First case of human babesiosis in Germany—Clinical presentation and molecular characterisation of the pathogen. Int. J. Med. Microbiol..

[5] Hildebrandt A., Hunfeld K.P., Baier M., Krumbholz A., Sachse S., Lorenzen T., Kiehntopf M., Fricke H.J., Straube E. (2007). First confirmed autochthonous case of human *Babesia microti* infection in Europe. Eur. J. Clin. Microbiol. Infect. Dis..

[6] Medlock J.M., Hansford K.M., Bormane A., Derdakova M., Estrada-Peña A., George J.C., Golovljova I., Jaenson T.G., Jensen J.K., Jensen P.M. (2013). Driving forces for changes in geographical distribution of *Ixodes ricinus* ticks in Europe. Parasites Vectors.

[7] Overzier E., Pfister K., Herb I., Mahling M., Bock G., Silaghi C. (2013). Detection of tick-borne pathogens in roe deer (*Capreolus capreolus*), in questing ticks (*Ixodes ricinus*), and in ticks infesting roe deer in southern Germany. Ticks Tick Borne Dis..

[8] Kauffmann M., Rehbein S., Hamel D., Lutz W., Heddergott M., Pfister K., Silaghi C. (2017). *Anaplasma phagocytophilum* and *Babesia* spp. in roe deer (*Capreolus capreolus*), fallow deer (*Dama dama*) and mouflon (*Ovis musimon*) in Germany. Mol. Cell Probes.

[9] Enigk K., Friedhoff K. (1962). *Babesia capreoli* n. sp. beim Reh (*Capreolus capreolus*). Zschr. Tropenmed. Parasitol..

[10] Duh D., Petrovec M., Bidovec A., Avsic-Zupanc T. (2005). Cervids as babesiae hosts, Slovenia. Emerg. Infect. Dis..

[11] Tampieri M.P., Galuppi R., Bonoli C., Cancrini G., Moretti A., Pietrobelli M. (2008). Wild ungulates as *Babesia* hosts in northern and central Italy. Vector Borne Zoonotic Dis..

[12] Silaghi C., Hamel D., Pfister K., Rehbein S. (2011). *Babesia* species and co-infection with *Anaplasma phagocytophilum* in free-ranging ungulates from Tyrol, Austria. Wien. Tierärztl Mschr..

[13] Zintl A., Finnerty E.J., Murphy T.M., de Waal T., Gray J.S. (2011). Babesias of red deer (*Cervus elaphus*) in Ireland. Vet. Res..

[14] Jouglin M., Fernández-de-Mera I.G., de la Cotte N., Ruiz-Fons F., Gortázar C., Moreau E., Bastian S., de la Fuente J., Malandrin L. (2014). Isolation and characterization of *Babesia pecorum* sp. nov. from farmed red deer (*Cervus elaphus*). Vet. Res..

[15] Michel A.O., Mathis A., Ryser-Degiorgis M.P. (2014). *Babesia* spp. in European wild ruminant species: Parasite diversity and risk factors for infection. Vet. Res..

[16] Zanet S., Trisciuoglio A., Bottero E., de Mera I.G., Gortazar C., Carpignano M.G., Ferroglio E. (2014). Piroplasmosis in wildlife: *Babesia* and *Theileria* affecting free-ranging ungulates and carnivores in the Italian Alps. Parasites Vectors.

[17] Pūraitė I., Rosef O., Radzijevskaja J., Lipatova I., Paulauskas A. (2016). The first detection of species of *Babesia* Starcovici, 1893 in moose, *Alces alces* (Linnaeus), in Norway. Fol. Parasitol..

[18] Hoby S., Robert N., Mathis A., Schmid N., Meli M.L., Hofmann-Lehmann R., Lutz H., Deplazes P., Ryser-Degiorgis M.-P. (2007). Babesiosis in free-ranging chamois (*Rupicapra r. rupicapra*) from Switzerland. Vet. Parasitol..

[19] Malandrin L., Jouglin M., Sun Y., Brisseau N., Chauvin A. (2010). Redescription of *Babesia capreoli* (Enigk and Friedhoff, 1962) from roe deer (*Capreolus capreolus*): Isolation, cultivation, host specificity, molecular characterisation and differentiation from *Babesia divergens*. Int. J. Parasitol..

[20] Stuen S., Olsson Engvall E., van de Pol I., Schouls L.M. (2001). Granulocytic ehrlichiosis in a roe deer calf in Norway. J. Wildl. Dis..

[21] Eick E. (2018). Das Sikawild. Eine Monographie.

[22] Gangl C. (2013). Hübsche kleine Japaner im Kommen. Jagd. Bayern..

[23] Helm G. (2013). Königsklasse Sikajagd. Jagd. Bayern.

[24] Weigl H. (2015). Kleiner Hirsch auf Abwegen I. Jagd. Bayern.

[25] Weigl H. (2015). Kleiner Hirsch auf Abwegen II. Jagd. Bayern.

[26] Dvořák J., Palyzová L. (2016). Analysis of the development and spatial distribution of sika deer (*Cervus nippon*) populations on the territory of the Czech Republic. Acta Univ. Agric. Silvic. Mendel. Brun..

[27] Silaghi C., Hamel D., Thiel C., Pfister K., Passos L.M., Rehbein S. (2011). Genetic variants of *Anaplasma phagocytophilum* in wild caprine and cervid ungulates from the Alps in Tyrol, Austria. Vector Borne Zoonotic Dis..

[28] Scharf W., Schauer S., Freyburger F., Petrovec M., Schaarschmidt-Kiener D., Liebisch G., Runge M., Ganter M., Kehl A., Dumler J.S. (2011). Distinct host species correlate with *Anaplasma phagocytophilum ankA* gene clusters. J. Clin. Microbiol..

[29] Hornok S., Sugár L., Fernández de Mera I.G., de la Fuente J., Horváth G., Kovács T., Micsutka A., Gönczi E., Flaisz B., Takács N. (2018). Tick- and fly-borne bacteria in ungulates: The prevalence of *Anaplasma phagocytophilum*, haemoplasmas and rickettsiae in water buffalo and deer species in Central Europe, Hungary. BMC Vet. Res..

[30] Robinson M.T., Shaw S.E., Morgan E.R. (2009). *Anaplasma phagocytophilum* infection in a multi-species deer community in the New Forest, England. Eur. J. Wildl Res..

[31] Ebani V.V., Cerri D., Fratini F., Ampola M., Andreani E. (2007). *Anaplasma phagocytophilum* infection in a fallow deer (*Dama dama*) population in a preserve of central Italy. New Microbiol..

[32] Rehbein S., Visser M., Jekel I., Silaghi C. (2014). Endoparasites of the fallow deer (*Dama dama*) of the Antheringer Au in Salzburg, Austria. Wien. Klin. Wochenschr..

[33] Wu D., Wuritu, Yoshikawa Y., Gaowa, Kawamori F., Ikegaya A., Ohtake M., Ohashi M., Shimada M., Takada A. (2015). A molecular and serological survey of Rickettsiales bacteria in wild sika deer (*Cervus nippon nippon*) in Shizuoka Prefecture, Japan: High prevalence of *Anaplasma* species. Jpn. J. Infect. Dis..

[34] Ybanez A., Matsumoto K., Kishimoto T., Inokuma H. (2012). Molecular analyses of a potentially novel *Anaplasma* species closely related to *Anaplasma phagocytophilum* detected in sika deer (*Cervus nippon yesoensis*) in Japan. Vet. Microbiol..

[35] Moustafa M.A.M., Lee K., Taylor K., Nakao R., Sashika M., Shimozuru M., Tsubota T. (2015). Molecular characterizaton and specific detection of *Anaplasma* species (AP-sd) in sika deer and its first detection in wild brown bears and rodents in Hokkaiko, Japan. Infect. Genet. Evol.

[36] Hapunik J., Víchová B., Karbowiak G., Wita I., Bogdaszewski M., Pet’ko B., Häselbarth K. (2011). Wild and farm breeding cervids infections with *Anaplasma phagocytophilum*. Ann. Agric. Environ. Med..

[37] Yang J., Liu Z., Niu Q., Luo J., Wang X., Yin H. (2017). Molecular detection of *Anaplasma phagocytophilum* in wild cervids and hares in China. J. Wildl. Dis..

[38] Silaghi C., Kohn B., Chirek A., Thiel C., Nolte I., Liebisch G., Pfister K. (2011). Relationship of molecular and clinical findings on *Anaplasma phagocytophilum* involved in natural infections of dogs. J. Clin. Microbiol..

[39] Silaghi C., Liebisch G., Pfister K. (2011). Genetic variants of *Anaplasma phagocytophilum* from 14 equine granulocytic anaplasmosis cases. Parasites Vectors.

[40] Nieder M., Silaghi C., Hamel D., Pfister K., Schmäschke R., Pfeffer M. (2012). Tick-borne fever caused by *Anaplasma phagocytophilum* in Germany: First laboratory confirmed case in a dairy cattle herd. Tierärztl. Prax. Ausg. G.

[41] Granquist E.G., Bårdsen K., Bergström K., Stuen S. (2010). Variant-and individual dependent nature of persistent *Anaplasma phagocytophilum* infection. Acta Vet. Scand..

[42] Huhn C., Winter C., Wolfsperger T., Wüppenhorst N., Strašek Smrdel K., Skuballa J., Pfäffle M., Petney T., Silaghi C., Dyachenko V. (2014). Analysis of the population structure of *Anaplasma phagocytophilum* using multilocus sequence typing. PLoS ONE.

[43] Jahfari S., Coipan E.C., Fonville M., van Leeuwen A.D., Hengeveld P., Heylen D., Heyman P., van Maanen C., Butler C.M., Földvári G. (2014). Circulation of four *Anaplasma phagocytophilum* ecotypes in Europe. Parasites Vectors.

[44] Aardeema M.L., von Loewenich F.D. (2015). Varying influences of selection and demography in host-adapted populations of the tick-transmitted bacterium, *Anaplasma phagocytophilum*. BMC Evol. Biol..

[45] Bonnet S., Jouglin M., L’Hostis M., Chauvin A. (2007). *Babesia* sp. EU1 from roe deer and transmission within *Ixodes ricinus*. Emerg. Infect. Dis..

[46] Elbaz E., Moustafa M.A.M., Lee K., Mohamed W.M.A., Nakao R., Shimozuru M., Sashika M., Younis E.E.A., El-Khodery S.A., Tsuboa T. (2017). Molecular identification and characterization of piroplasm species in Hokkaido sika deer (*Cervus nippon yesoensis*), Japan. Ticks Tick BBMorne. Dis..

[47] Liu J., Yang J., Guan G., Liu A., Wang B., Luo J., Yin H. (2016). Molecular detection and identification of piroplasms in sika deer (*Cervus nippon*) from Jilin province, China. Parasites Vectors.

[48] He L., Khan M.K., Zhang W.-J., Zhang Q.-L., Zhou Y.-Q., Hu M., Zhao J. (2012). Detection and identification of *Theileria* infection in sika deer (*Cervus nippon*) in China. J. Parasitol..

[49] Li Y., Chen Z., Zhijie L., Liu J., Yang J., Li Q., Li Y., Cen S., Guan G., Ren Q. (2014). Molecular identification of *Theileria* parasites of northwestern Chinese Cervidae. Parasites Vectors.

[50] Zamoto-Niikura A., Tsuji M., Imaoka K., Kimura M., Moirkawa S., Holman P., Hirata H., Ishihara C. (2014). Sika deer carrying *Babesia* parasites closely related *B. divergens*, Japan. Emerg. Inf. Dis..

[51] Gray J.S., Murphy T.M., Waldrup K.A., Wagner G.G., Blewett D.A., Harrington R. (1991). Comparative studies of *Babesia* spp. from white-tailed and sika deer. J. Wildl. Dis..

[52] Plötz C., Rehbein S., Bamler H., Reindl H., Pfister K., Scheuerle M.C. (2015). *Fascioloides magna* epizootiology in a deer farm in Germany. Berl. Münch. Tierärztl. Wochenschr..

[53] Rehbein S., Visser M., Plötz C., Schwarz L., Lindner T., Bamler H., Pfister K. (2019). Endoparasites of red deer (*Cervus elaphus*) from a deer farm endemic with fascioloidosis in Germany. Berl. Münch. Tierärztl. Wochenschr..

[54] Courtney J., Kostelnik L.M., Zeidner N.S., Massung R.F. (2004). Multiplex real-time PCR for detection of *Anaplasma phagocytophilum* and *Borrelia burgdorferi*. J. Clin. Microbiol..

[55] Massung R.F., Slater K., Owens J.H., Nicholson W.L., Mather T.N., Solberg V.B., Olson J.G. (1998). Nested PCR assay for detection of granulocytic ehrlichiae. J. Clin. Microbiol..

[56] Casati S., Sager H., Gern L., Piffaretti J.C. (2006). Presence of potentially pathogenic *Babesia* sp. for human in *Ixodes ricinus* in Switzerland. Ann. Agric. Environ. Med..

